# Developing, Implementing, and Evaluating an Artificial Intelligence–Guided Mental Health Resource Navigation Chatbot for Health Care Workers and Their Families During and Following the COVID-19 Pandemic: Protocol for a Cross-sectional Study

**DOI:** 10.2196/33717

**Published:** 2022-07-25

**Authors:** Jasmine M Noble, Ali Zamani, MohamadAli Gharaat, Dylan Merrick, Nathanial Maeda, Alex Lambe Foster, Isabella Nikolaidis, Rachel Goud, Eleni Stroulia, Vincent I O Agyapong, Andrew J Greenshaw, Simon Lambert, Dave Gallson, Ken Porter, Debbie Turner, Osmar Zaiane

**Affiliations:** 1 Department of Computing Science University of Alberta Edmonton, AB Canada; 2 Department of Psychiatry Faculty of Medicine and Dentistry University of Alberta Edmonton, AB Canada; 3 Alberta Machine Intelligence Institute Edmonton, AB Canada; 4 Department of Indigenous Studies University of Saskatchewan Regina, SK Canada; 5 Rehabilitation Robotics Lab Faculty of Rehabilitation Medicine University of Alberta Edmonton, AB Canada; 6 Mood Disorders Society of Canada Ottawa, ON Canada; 7 Temerty Faculty of Medicine University of Toronto Toronto, ON Canada; 8 Department of Psychiatry Faculty of Medicine Dalhousie University Halifax, NS Canada; 9 Asia-Pacific Economic Cooperation Digital Hub for Mental Health Vancouver, BC Canada; 10 Network Environments for Indigenous Health Research National Coordinating Centre Saskatoon, SK Canada

**Keywords:** eHealth, chatbot, conversational agent, health system navigation, electronic health care, mobile phone

## Abstract

**Background:**

Approximately 1 in 3 Canadians will experience an addiction or mental health challenge at some point in their lifetime. Unfortunately, there are multiple barriers to accessing mental health care, including system fragmentation, episodic care, long wait times, and insufficient support for health system navigation. In addition, stigma may further reduce an individual’s likelihood of seeking support. Digital technologies present new and exciting opportunities to bridge significant gaps in mental health care service provision, reduce barriers pertaining to stigma, and improve health outcomes for patients and mental health system integration and efficiency. Chatbots (ie, software systems that use artificial intelligence to carry out conversations with people) may be explored to support those in need of information or access to services and present the opportunity to address gaps in traditional, fragmented, or episodic mental health system structures on demand with personalized attention. The recent COVID-19 pandemic has exacerbated even further the need for mental health support among Canadians and called attention to the inefficiencies of our system. As health care workers and their families are at an even greater risk of mental illness and psychological distress during the COVID-19 pandemic, this technology will be first piloted with the goal of supporting this vulnerable group.

**Objective:**

This pilot study seeks to evaluate the effectiveness of the Mental Health Intelligent Information Resource Assistant in supporting health care workers and their families in the Canadian provinces of Alberta and Nova Scotia with the provision of appropriate information on mental health issues, services, and programs based on personalized needs.

**Methods:**

The effectiveness of the technology will be assessed via voluntary follow-up surveys and an analysis of client interactions and engagement with the chatbot. Client satisfaction with the chatbot will also be assessed.

**Results:**

This project was initiated on April 1, 2021. Ethics approval was granted on August 12, 2021, by the University of Alberta Health Research Board (PRO00109148) and on April 21, 2022, by the Nova Scotia Health Authority Research Ethics Board (1027474). Data collection is anticipated to take place from May 2, 2022, to May 2, 2023. Publication of preliminary results will be sought in spring or summer 2022, with a more comprehensive evaluation completed by spring 2023 following the collection of a larger data set.

**Conclusions:**

Our findings can be incorporated into public policy and planning around mental health system navigation by Canadian mental health care providers—from large public health authorities to small community-based, not-for-profit organizations. This may serve to support the development of an additional touch point, or point of entry, for individuals to access the appropriate services or care when they need them, wherever they are.

**International Registered Report Identifier (IRRID):**

PRR1-10.2196/33717

## Introduction

### Background and Rationale

Mental disorders are the leading cause of disability in Canada; approximately 1 in 3 Canadians will experience substance use or mental health disorders in their lifetime [1,2]. Unfortunately, there are also significant gaps in care. According to a 2018 study, 5.3 million Canadians expressed a need for mental health services in a 12-month period [3]. Of these, 43.8% reported that their mental health needs were not being adequately met [3]. Of those reporting unmet or only partially met needs, 78.2% identified personal circumstances, including affordability and not knowing where to receive help, as barriers to care [3].

Barriers to seeking support include stigma, denial, concerns over privacy, and difficulty connecting effectively with a care provider [4-7]. In addition, prominent access issues include fragmented or episodic care, lack of support for navigating the health care system and connecting with an appropriate provider or specialist, and long wait times to access services [4-10].

Canada’s publicly funded health care system is administered and delivered by the provinces and territories through public health authorities or entities operating on a nonprofit basis. Hospitals and other health care services deemed medically necessary must be insured by provincial and territorial plans. Many citizens acquire additional private insurance to pay for unfunded services [11]. Mental health care coverage across Canada varies widely, and many available services are not deemed medically necessary despite mental health being increasingly recognized as fundamental to health. Only mental health services received in hospital settings are covered universally by Canada’s public health system. Mental health care in Canada is unique as it is provided by a “meshwork” of local hospitals, community programs, residential care centers, private practices, and more [12]. Adding to this complexity, many organizations are particular to 1 jurisdiction or specific to a certain type of mental health concern.

Canada’s system has been described as a “labyrinth” where individuals may even resort to paying private sector agents to act on their behalf to find and connect with services, further exacerbating socioeconomic inequalities in access to care [13]. Many Canadians who have received unsatisfactory help for their mental health needs reported “not knowing where to go” as a primary barrier to care [14]. Testimonies of Ontario-based patients and caregivers highlight feelings of confusion in having to navigate this system on their own, resulting in longer delays in care access [15]. Wait times have been as long as 2 and a half years [14], with many individuals receiving no documented care [10]. The Wait Time Alliance 2014 Report Card highlights lack of system coordination and insufficient staff and resources as determinants of long wait times to access mental health services in Canada [15]. Heightened demands for care and lack of navigation toward community services contribute to overcrowding within emergency departments, with a 75% increase in mental health–related visits for patients aged 5 to 24 years since 2006 [16]. System integration and system navigation support services between community-based health and social services and formal health care providers have been identified as a key policy issue in Canada and other jurisdictions such as the United Kingdom [16-23], where lack of knowledge of service options often poses a barrier to referrals from health care providers to community-based services [18,23-26].

### The Impact of the COVID-19 Pandemic on Mental Health

In 2019, an outbreak of COVID-19 (SARS-CoV-2) resulted in a global pandemic. By early 2022, COVID-19 had spread worldwide, with >334 million known cases and >5.5 million deaths [27]. In anticipation of a high volume of serious hospitalizations with technical respiratory needs, Canadians were asked to self-quarantine or practice social distancing to reduce the burden on health systems [28]. This intensified the mental health crisis within Canada; according to an Angus Reid Institute poll, 50% of Canadian respondents indicated that their mental health had worsened over the COVID-19 pandemic, with 10% indicating that it had worsened “a lot” [29]. Multiple public surveys deployed during the pandemic reported respondents’ experiences of multiple mental health stressors such as economic instability, fear of becoming sick, and life disruption as a cause of the COVID-19 pandemic, resulting in stress, anxiety, and depression [30]. A recent Ontario survey revealed that approximately 25% of respondents reported unmet mental health needs as a result of the pandemic, moderate to severe anxiety, and symptoms of loneliness and depression [31].

In accordance with the negative mental health outcomes observed in this and previous epidemics and pandemics [32,33], it is widely agreed by the international medical community that a wave of widespread need for mental health–related services will result from the pandemic that will persist beyond the acute phase [30]. Within the Canadian context, in consideration of the prepandemic prevalence of mental illnesses such as depression (lifetime prevalence of 5% in Canadian men and 10% in Canadian women [34]) and insomnia (12-month prevalence ranging from 9.5% to 24% [35-38]) and existing gaps in service delivery, public health practitioners and policy leaders must urgently consider innovative ways to connect a large portion of the Canadian public with appropriate services in an efficient manner.

In addition to the negative impact on Canadians’ mental health, many services have faced disruptions because of adjusting to social distancing and capacity restrictions, often eliminating face to face in lieu of remote service settings [39]. Many countries have developed new web-based mental health information sites or phone lines to provide coping support [40]. For those facing modest mental health burdens, connection with these web-based resources can aid in self-management and may provide a bridge before professional support is available [39]. With these changes in offered services and increased web-based application use, navigation to individual personalized, timely, and relevant resources is increasingly important.

### The Mental Health of Health Care Workers

Health care workers and their families are particularly vulnerable during pandemics and, in reflection of anticipated needs, are the target participant group for this pilot study. Health care workers face an increase in mental health risk factors, including anxiety, burnout, and depression, because of factors such as increased exposure and risk of disease transmission to themselves or others (eg, family and friends) and unsafe (eg, personal protective gear shortages) or stressful working conditions [38]. Of concern is the trauma that health care workers witness within the workplace, how their ongoing work limits their ability to address their own mental health concerns, and how they may be processing these experiences when they are outside of the workplace with more time to reprocess what they see. For example, a recent umbrella review of meta-analyses found that the prevalence of anxiety and depression among health care workers was relatively high at 24.94% [39]. A recent survey by the Canadian Centre for Addiction and Mental Health (January 2022) documented an increase in self-reported symptoms of severe anxiety (37% compared with 23.5% in summer 2021) and depression (35.7% compared with 24.8% in summer 2021) among health care workers and other frontline workers [31], suggesting that mental health problems are being exacerbated with time. Together, these risk factors may lead to health care workers resigning from their positions, increasing staff shortages and, in turn, pressures on the remaining employees [40]. On the basis of our findings within this pilot group, we aim to further refine, scale, and spread the implementation of our chatbot to be used by Canada’s general public.

### Opportunities for Health Chatbots

Digital technologies provide an opportunity to bridge service gaps, increase points of access to and knowledge of the mental health care system and existing services, enhance mental health literacy, and permit greater health system and social system integration, which could improve health and social system coordination, efficiency, patient navigation, satisfaction, and overall health outcomes [41]. In addition, efficiencies realized through the use of new technology may lower health care costs, enabling resources to be redirected to other areas of priority. Artificial intelligence (AI) presents the opportunity to bypass barriers inherent to traditional brick-and-mortar health system structures, meet individuals in need in a discrete and personalized way, and connect them with services in a timely manner regardless of where they are. For example, commonly cited factors identified for why individuals choose to access web-based services include 24-hour accessibility, ease of accessibility despite geographic location, anonymity, and privacy [42-45]. Although further analysis is required in the context of mental health care, research suggests that patients report greater comfort or preference in disclosing sensitive health information to a computer or technological device than to a human [46,47]. AI then presents the opportunity to also address social stigma as a barrier to care, which may hinder an individual’s drive or motivation to seek access to care.

Chatbots can be defined as computer programs that use AI methods, including natural language processing (NLP) and machine learning, to simulate conversations with human users. Existing evidence supports the use of health chatbots for empowering users to engage in physical activity and consumption of nutritious food and increasing patient access to health information, among other benefits [48-51]. Although human-computer interaction technology itself is not new as a concept, evaluative research on the use of applied AI as a tool for bridging gaps in mental health care is limited. More specifically, although chatbots currently show promise in a variety of health care settings [52-54], there is limited information on their effectiveness in supporting mental health system navigation [52,55-57]. As such, the use of a conversational chatbot for this general purpose is novel. In addition, existing chatbots are commonly tailored to address one or a limited range of mental health issues [58]. Our conversational chatbot, the Mental Health Intelligent Information Resource Assistant (MIRA), seeks to support a wide range of mental health disorders and considerations.

Most research to date has evaluated constrained client input (options that are provided to the client for input), and research on unconstrained natural language opportunities remains in its infancy [56]. Chatbots in mental health have been characterized or criticized as being predominantly rule-based (chatbot-led and controlled vs user-controlled) and are offered as stand-alone software (vs web-based software, complicating ease of client access). MIRA is a web-based, hybrid NLP and decision tree user-controlled AI chatbot. In this context, these features are novel in their application to the mental health space. See the *Methods* section for more details on each of these design elements.

With advances in dialogue management and conversational flexibility enabled through the establishment of complex neural networks that include sentiment analysis, chatbots within the space of mental health have the opportunity to play an important role in patient care.

Fortunately for the emergence of digital health intervention options, the uptake of technology among the general public has been substantive. There are >3.96 billion internet users internationally [59]. In Canada, 91% of the population is estimated to be actively using the internet, and 85% have a cell phone (65% have a smartphone specifically) [60,61]. As such, there remain significant opportunities to use existing and widely adopted technological infrastructure to bridge significant gaps in care and improve health outcomes for Canadians.

### The MIRA Project

In this paper, our pan-Canadian, multidisciplinary team of subject matter experts, including individuals with lived experience, members of the Indigenous community, clinicians, and psychiatry and computing science experts, report on the design, implementation, and anticipated evaluation of MIRA, a domain-specific AI-enabled chatbot able to understand common taxonomies in the mental health domain and respond with relevant, appropriate resources aligned with the clients’ intents and needs. The MIRA chatbot is an informational chatbot only and does not provide medical advice (ie, it does not diagnose or provide treatment recommendations), nor does it replace a counselor or mental health professional. The population group of interest for this pilot were health care workers and their families in the Canadian provinces of Alberta and Nova Scotia.

In total, 2 additional components have been developed to complement the chatbot’s functionality, including a resource management portal (the MIRA Resource Portal) and a Selenium (Software Freedom Conservancy)-based [62] automated testing framework. MIRA does not search for resource recommendations extracted from the open internet. Instead, MIRA draws recommendations from the MIRA Resource Portal, which not only facilitates the input and expert validation of mental health resources for use by the chatbot but also automatically monitors validated resources for any changes after approval and subsequently reports them to the editors. Our Selenium-based testing framework uses AI to automatically generate diverse wording test cases to assess the chatbot with different dialogue flows using diverse wordings and intentional minor spelling errors.

This study will investigate the effectiveness of MIRA in its ability to successfully connect health care workers and their families with appropriate information on mental health issues and local services and programs based on their identified needs. The effectiveness of the technology will be evaluated primarily through data collected via voluntary follow-up surveys and client interactions and engagement with the chatbot. Client satisfaction with the chatbot will also be assessed. We hypothesize that the chat will successfully connect users with appropriate health resources (eg, mental health educational resources, the Mood Disorders Society of Canada [MDSC] peer support program, posttraumatic stress disorder training, and web-based peer support; see the *Outcome Evaluation* section for more details).

## Methods

The following subsections describe how the chatbot was developed and implemented and how it will be subsequently evaluated.

### Chatbot Development

#### A Multidisciplinary Team

In recognition of the complex nature of developing and implementing accepted and effective state-of-the-art computing science technologies seeking to support mental health and wellness within the public health domain, a multidisciplinary team is required.

The MIRA Operational Team (including senior leadership, fellows, students, and support staff) and voluntary Expert Advisory Committee include computing science and psychiatry experts, health care workers, and family members and individuals with lived experience. To develop a new technology that is accessible to all Canadians and does not perpetuate the systemic racism inherent within the public health system, MIRA is being cocreated with the Indigenous community and includes an ethnically and culturally diverse team leading and supporting its development from tip to tail. See Multimedia Appendix 1 for a graphical description of this multidisciplinary approach.

#### Developing MIRA

MIRA was built via Rasa Open Source (Rasa Technologies Inc), an open-source conversational AI platform [63], as team members had familiarity with the platform and it was considered advantageous for the implementation of advanced NLP; owing to its flexibility and ease of integration; and because it was deemed most customizable by our chatbot team in comparison with other platforms such as Botkit, BotPress, MindMeld, and DeepPavlov [64-67]. Its customization would also allow for the incorporation of progressively more complicated or advanced forms of AI and NLP. This is imperative for future iterations of MIRA (beyond this pilot), where the study team intends to program the chatbot to adapt its behavior differently depending on the geographic and linguistic context of the individual using the chatbot or interface. This approach, using base code from Rasa Open Source and enhancing, adding to, and adapting it based on our needs for this project, allows our team to both pilot a state-of-the-art viable product in a reasonable time frame to address an urgent public health need and ensure that more advanced computing science techniques and developments can be incorporated incrementally and tested over several years to further enhance the chatbot capabilities. This also builds on lessons learned from other researchers developing similar technologies in health, where deploying a “working solution” at the time was done at the expense of “...more innovative and potentially better solutions...” [68].

MIRA was built as a web-based chatbot (vs a stand-alone chatbot) to increase accessibility to it as no installation of software or applications is needed to use the chatbot and it can be used on any device regardless of the operating system. This also builds on lessons learned from other research in the field, where stand-alone chatbots were predominantly used [58].

The development of MIRA was guided by a chart-flow diagram via Lucidchart (Lucid) [69], a web-based collaborative design platform. This conversational flow diagram was initially developed by a psychiatry expert (JMN) on our team and then tested and refined by study team members from MDSC, an organization led by individuals with lived experience, including the categorization of anticipated client *intents* (what the client would like to accomplish) and appropriate chatbot *utterances* (the chatbot’s responses to a client). Following this preliminary structuring, our computing science team members built the MIRA chatbot to follow the chart-flow diagram as a guide. To further refine the chatbot, the multidisciplinary team was asked to imagine hypothetical questions that could be received by the chatbot. Approximately 200 hypothetical questions were developed (133 distinct questions along with variations in question types). The questions were subsequently organized into *intents*. The chatbot was initially trained on these *intents*. When new intents were revealed, the chart-flow diagram was adjusted to include them. Where possible, *intents* and *utterances* were enhanced using existing open-source libraries on chatbot dialogue. All chatbot *utterances* were reviewed by members of the team from the MDSC to ensure that the language used was at an appropriate reading level, clear, considerate, and respectful. The rationale for approaching the chatbot design and training data in this manner was in reflection of the complicated nature of mental health. The chatbot needed to be tailored to ensure that it behaved in a way that would guide the user to resource recommendations appropriately, effectively, and respectfully. Lucidchart allowed non–computing science team members (eg, psychiatry and lived experience experts) to communicate necessary chatbot conversational flow behaviors to the computing science team clearly and effectively, including any emergency- or urgency-related prompts and responses. The open-source use of big data has been significantly criticized for perpetuating systemic racism and societal inequalities [70]. As such, developing training data using our multidisciplinary team was important to ensure that chatbot behavior remained respectful and reduce existing issues inherent in big data. Furthermore, chatbot behavior was reviewed by members of the team with lived experience to ensure that the chatbot responses were trauma-informed to not exacerbate trauma or challenges individuals accessing services may face.

**Figure 1:**
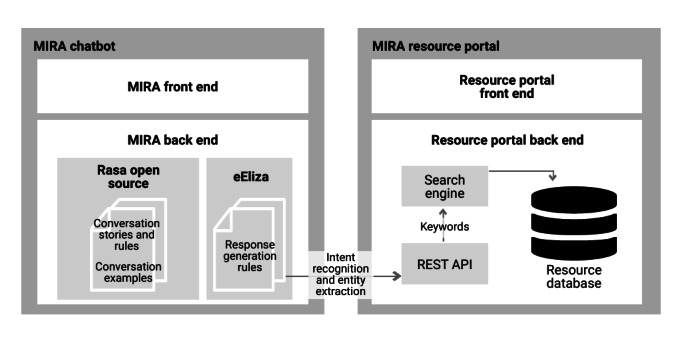
Architectural diagram of the Mental Health Intelligent Information Resource Assistant (MIRA) chatbot and resource portal system. API: application programming interface; REST: Representational State Transfer.

At the request of the MDSC, the computing science team incorporated additional conversational functionality reflective of ELIZA [71], an early NLP conversational AI algorithm, to add nonscripted responses. This was to reduce the transactional or robotic feel or experience a client had with the chatbot and allow a client to speak freely with the chatbot before asking for assistance in finding specific resources. For the preliminary design, the computing science team adjusted the ELIZA code to include entity extraction (this adjusted code is referred to as *eEliza*, where *e* denotes *enhanced*, and can be found in Figure 1). The extracted entities are then used in conversation back to the client (to acknowledge the information shared by the client) as well as to refine resource recommendations to be provided later in the interaction. The use of Generative Pre-trained Transformer [72], an open-source AI algorithm that translates input from clients and generates at times human-like output, is currently being explored to further enhance the conversational functionality of the chatbot for this pilot phase.

Of importance is the use of both NLP (via text-based entry) and decision trees (rule-based responses based on the use of predetermined button options) to support response generation. Most chatbots in mental health use either one or the other, whereas MIRA uses a hybrid approach to enhance functionality and end-user experience by offering them the choice or control to use both interchangeably during their exchange with MIRA. It is our understanding at the time of writing this paper that this approach in itself (using NLP and rule-based responses and allowing users to control the conversation) is a novel application of AI within the mental health field [58].

The chatbot was built to associate various terms or phrases as being of an urgent or serious nature and has been prompted to immediately provide information on emergency services should this association be triggered. For example, if the client indicates that they are experiencing suicidal thoughts, the chatbot will immediately provide them with emergency contact information and the Crisis Services Canada phone number, chat line, and texting information. This emergency response was flagged by mental health experts on our team and our Expert Advisory Committee as being of critical importance in supporting individuals experiencing urgent mental health–related challenges. Hypothetical phrases used for baseline training data were developed with the assistance of these members as well.

Following this work, our computing science team members worked on the incorporation of data augmentation (ie, the use of an AI algorithm that, using existing examples and hypothetical questions, can create new variations of hypothetical questions that could be asked to further refine chatbot functionality) as well as web browser automation via the Selenium platform to test chatbot functionality to mimic anticipated and unanticipated client questions, responses, and behaviors. Over 20 different data augmentation algorithms were used, including contextual word embeddings, random character errors, and synonym augmentations. Stanford’s CoreNLP (Stanford NLP Group), an open-source NLP tool, will be used to further enhance entity extraction [73].

The two main purposes of MIRA are to provide individuals with (1) information or education on substance use and mental illnesses generally (simple educational information including general definitions and descriptions of symptoms written at a lay audience level) and (2) information on services and programs in Canada or, if voluntarily provided, in the specific city or village, region, or province of the identified end user. The level of specificity of the information, services, or programs provided is dependent on the level of information voluntarily shared by the client. For example, the client may ask for a definition of mood disorders or, alternatively, for an in-person group therapy program for individuals managing major depressive disorder in Edmonton, Alberta. The programming of these 2 components requires different approaches, as detailed below.

The education resource component of the chatbot was developed by first identifying common mental health ailments or challenges. A list of psychiatric disorders was extracted from the Diagnostic and Statistical Manual of Mental Disorders by a psychiatry expert on the team, who then extracted common language used on the web by reputable (known government and not-for-profit, government-supported) organizations whose websites were tailored to a lay audience and linked clinical and common language. This linking was helpful both to train the chatbot on relationships between lexicons used in the psychiatric field and audiences and to serve as a preliminary list of common ailments. Following the development of these lists, volunteers at the MDSC supported the collection of web-based resources that provided the following information on each specific ailment: definition of the disorder or ailment, list of associated symptoms, and a description of common associated treatments. These web-based resources and their relevant informational data were individually logged in the MIRA portal (MIRA portal details outlined below) and subject to review by a 30-member Expert Advisory Committee.

The service and program resource component was developed by first logging a resource list provided on the MDSC website. The act of logging was conducted by MDSC volunteers. Following this, government websites were reviewed by team members and volunteers for additional resource lists. Where lists of recommended resources were provided, volunteers were asked to log these resources. These web-based resources were also subject to review by a 30-member Expert Advisory Committee. The MIRA portal currently has >750 fully vetted resources.

#### Testing MIRA

The MIRA core team collaboratively determined a quality threshold for the release of a minimum viable chatbot prototype based on a variety of performance metrics. Performance measures, including accuracy of intent recognition and entity extraction as well as average rending time, were used by the computing science team to monitor the progress of chatbot training and refinement. Where needed, the team strategically addressed particular elements of the chatbot that they felt may enhance measures reporting lower than acceptable metrics.

The chatbot was in alpha testing from December 2021 to February 2022. The chatbot was then beta tested by members of our 30-member, multidisciplinary Expert Advisory Committee from February 7, 2022, to May 1, 2022. Comments were logged, and changes were made to the chatbot in real time. Testing was enhanced using automation via Selenium, a web browser automation tool, to repeatedly test anticipated end-user behavior and log programming bugs and errors.

The chatbot was launched for the pilot population on May 2, 2022. Data collection is anticipated to take place from May 2, 2022, to May 2, 2023. The publication of a preliminary synthesis of the results will be sought in spring or summer 2022, followed by a more comprehensive evaluation in spring 2023.

#### Interacting With MIRA

Once the client clicks on the MIRA URL [74], they are directed to a webpage with the MIRA chatbot interface. MIRA begins by welcoming the client and asks them for their consent to use anonymized data from the conversation to evaluate and improve its services, with a link to a pop-up with consent information (Multimedia Appendix 2). If the client provides consent, the chatbot then asks a short series of demographic-related questions—employment type, location, and end user (eg, for the client or for someone else; if *someone else*, then the age of the end user is also collected; Multimedia Appendix 3). Following these preliminary questions, the chatbot asks in an open-ended manner (eg, “How can I help you?”) and provides some examples of questions that could be asked in the form of button options (eg, “I want to find programs and services” or “I want to learn coping skills”; Multimedia Appendix 4). If a client asks to “chat” with MIRA or begins expressing feelings opposed to an obvious request for information, eEliza programming will be prompted (Multimedia Appendix 5). The MIRA website, MIRA chatbot, and MIRA Resource Portal (renamed the MIRA Resource Library for the May 2 launch) were tested for compatibility with multiple electronic devices, including tablets, smartphones, and desktop computers, as well as multiple browser applications, including Safari and Chrome.

#### The MIRA Portal

The internet may contain information with inaccurate content, bias, and insufficient evidence [75,76]. This is why MIRA does not draw resource recommendations via the open internet and, instead, provides resource recommendations drawn from the MIRA Resource Portal. The MIRA Resource Portal is a resource repository in which MDSC volunteers have cataloged and annotated metadata of >750 resources to date. These resources were assessed for quality by a 30-member Expert Advisory Committee using an approach reflecting the academic peer-review process. More specifically, resources were assigned to committee members by an editor (the study coordinator). Each resource was subject to review by at least two different reviewers. Reviewers were guided by an evaluation matrix developed using a hybrid of items that were drawn from existing validated tools [75,77,78]. The factors assessed included (1) readability, (2) accessibility, and (3) quality [75,77,78]. This hybrid approach was taken as our definition of *resource* was broad (see the *Resource Types* section) and included many types of resources (eg, phone number, website links, videos, audio recordings, images, and apps) and, as such, to our knowledge, no validated tool existed at the time of writing this paper. To date, >1600 resource reviews have been conducted, resulting in 750 resources that have been fully vetted and approved by the Expert Advisory Committee and are now accessible by the chatbot.

For the purposes of this study, *resource* is defined as evidence-supported, relevant, and reliable information that would satisfy an end user of the chatbot in retrieving general mental health information or connecting with the appropriate mental health services for their identified needs and circumstances. A recommended resource can be provided at the end of a dialogue as the final outcome or at any time during the exchange.

#### Resource Types

The types of resources included in the MIRA Resource Portal are shown in Textbox 1.

**Types of resources included in the Mental Health Intelligent Information Resource Assistant Resource Portal.**

**Resource types**

- Health system navigation: information that connects clients with programs and services—includes a textual description of the resource and any of the elements of a typical contact record (email address, phone number, physical address of in-person service, hours of operation, and URL if it is a website)
- Informational or education reference: includes a textual description of the resource and the website URL or the URL to an audio, video, image, or PDF attachment
- Simple answer: just a textual description that can be provided as an answer to a direct factual question

If a resource received a mixed review, it was then subject to review by a third reviewer (a tiebreaker who was also a member of the Expert Advisory Committee). Reviews were made anonymous to everyone with the exception of the editor, who was responsible for assigning resources, to allow reviewers to be candid, and all reviewers were asked to acknowledge any conflicts of interest before being permitted to review a resource. See Figure 1 for an architectural diagram of the MIRA chatbot and portal system.

### Participants

For the purposes of this project, the definition of the target sample was broadened to capture all health-related personnel and their respective families who may be affected by heightened mental health burden in light of the ongoing pandemic. In addition, direct or vicarious trauma or psychological distress in a health care environment is not limited to medical staff [79,80]. Therefore, *health care worker* will be defined as outlined in Textbox 2.

**Definition of *health worker* for this study.**

***Health worker* definition**

- “Any health professionals and any staff member, contract worker, student or trainee, registered volunteer, or other essential caregiver currently working in a healthcare organization, including workers that are not providing direct patient care and are frequently in the patient environment. This includes cleaning staff, food services staff, information technology staff, security, research staff, and other administrative staff.
- Workers providing healthcare service or direct patient service in a congregate, residential or community setting outside of a healthcare organization (e.g., nurse providing patient care in a school, worker performing personal support services in an assisted living facility, medical first responder in the community, peer worker in a shelter)” [81].

The definition of health care worker varies considerably by health authority or administrator. As such, the following numbers were used to determine a general goal post for a target sample size recognizing that the data sources (Alberta Health Services and Nova Scotia Health Authority) may have differing inclusion criteria for what is categorized as a health care worker. There are approximately 240,000 frontline Alberta Health Services workers in Alberta [82], and there are 23,400 health care workers in Nova Scotia [83]. To include family members into sample size calculations, 2011 Statistics Canada estimates on the average number of individuals in a household were used (2.5 per household) [84]. With a 95% CI and 3% margin of error, the sample size target was estimated to be 1066 for Alberta and 1048 for Nova Scotia. More specifically, this sample size refers to the number of participants who consent to using the chatbot. This study will seek to slightly oversample from each province (Alberta: n=1100; Nova Scotia: n=1100). In reflection of the general experiences of other researchers with participation rates for web-based surveys (34%-43% [85-89]), we will aim to collect between 374 and 473 partial and complete baseline surveys and 110 partial and complete follow-up surveys per province. To the authors’ knowledge at the time of writing this paper, this study is the first to evaluate the use of conversational agents or chatbots to support mental health system navigation; as such, the effect size is unknown at the time of the design phase of the study, and power analysis will need to be conducted as the study team actively gathers data to ensure that our sample size is adequate. Therefore, the sample size is subject to re-estimation during the course of this study.

Participant recruitment will be conducted through snowball sampling via word of mouth, social media advertisements, physical advertisements (study posters and study information posted on physical bulletin boards in hospital staff rooms as well as in newsletters to staff where possible), and referrals via our network and partners (including medical professional organizations). After consenting to take part in the study via a web-based consent form, the participants will be asked if they would like to take part in voluntary follow-up surveys following the use of the chatbot. Regardless of their response, they will then be able to immediately engage with the chatbot. The inclusion criteria are being aged >18 years at the start of the study, being a health care worker or a family member of a health care worker, being located in Alberta or Nova Scotia, being able to speak and read English, and having access to the internet. The chatbot will be developed with the option of adding French and other languages of peoples geographically located within the settlement of Canada in future iterations of the chatbot (eg, Cree, Inuktitut, and Ojibway). The exclusion criteria are individuals aged <18 years, individuals in provinces outside Alberta and Nova Scotia, those with limited comprehension of English, and those without internet access. However, if a client tries to access this service to support an individual aged <18 years or outside the 2 provinces indicated, the chatbot will include cross-Canadian resources that could support them.

### Procedures

This project was initiated on April 1, 2021. Data collection began on May 2, 2022, and will continue until May 2, 2023. The publication of preliminary results will be sought following the synthesis of data in spring or summer 2022. A final report will be developed in spring 2023.

Health care workers from the provinces of Nova Scotia and Alberta will be invited to use the chatbot service. Family members of health care workers will also be welcome to participate in the study. Participant recruitment will be conducted via snowball sampling through word of mouth, social media advertisements, physical advertisements, and referrals via our network and partners (including medical professional organizations). Potential participants will be asked to provide informed consent before receiving services from the virtual assistant. Although the participants will be encouraged to register their email address so that the study team can send them voluntary follow-up surveys to evaluate program performance (more on the surveys below), registration will be voluntary. Regardless of registration, the participants will be provided with access to MIRA following the provision of consent.

If a participant registers their email address, they will be provided with 2 voluntary surveys: one at baseline (immediately following use of the chatbot) and a second one at 24 hours following the initial use of the chatbot. The surveys will collect demographic information (eg, year of birth, gender identity, and visible minority status); ask the participants whether they followed through with a recommended resource and the perceived appropriateness of that resource (eg, “Were the resource(s) that MIRA recommended to you during your conversation appropriate?” or “After your conversation with the MIRA, did you follow-up or connect with the resource(s) that the chatbot recommended?”); and assess baseline mental and physical health via the Clinical Outcomes in Routine Evaluation System (CORE-10), a brief, validated 10-item assessment and outcome measurement tool used to assess conditions including anxiety, depression, physical problems, and risk to self [90]. Select items from the Embodied Conversational Agent Trust Questionnaire (ETQ)—for example, “Did you feel that MIRA was competent?” (4-point Likert scale from 0=not at all to 3=completely)—and Acceptability E-scale (AES)—for example, “How much do you agree with the following statements? MIRA gave me information that was relevant to my concern” (5-point Likert scale from “strongly agree” to “strongly disagree”)—are used to assess client satisfaction and acceptability, including perceived usability, benevolence, credibility, and trustworthiness [91].

In addition to the voluntary survey, data will be extracted in aggregate of the general use of the technology to assess effectiveness and engagement, including topics most frequently raised, average time spent on the service, number of resources provided in an average conversation, number of client interactions with links, most frequent recommendations by the chatbot to clients, number of resources recommended by the chatbot in an average conversation, average number of objections raised by clients in conversations, and intent identification and entity extraction accuracy. The use of aggregate data in this manner will be flagged in the consent form preceding the use of the virtual assistant. Participation on the platform will remain anonymous with the exception of the satisfaction surveys (voluntary), which may be temporarily linked via email address to track survey responses at different time intervals (baseline and 24 hours). Following linkages, emails will be permanently deleted and replaced with a randomized participant number to further protect anonymity. Transcripts between clients and the chatbot will be used anonymously to further train and refine the chatbot. The transcripts do not contain any identifiable information and will only be used to improve chatbot functionality. The use of transcripts by the chatbot to serve as a form of memory from which the chatbot will learn and teach itself to perform in a more refined manner is outlined in the consent form provided to the participants. To further protect anonymity, the chatbot has been programmed to remove any personally identifiable information from the transcripts before saving them (eg, if a name is provided, it is omitted from the saved transcript).

There is no standardized approach to evaluate chatbots within the field of health [56,92]. To determine which variables to collect for analysis, our team aggregated findings from several academic studies and reviews that described the technical characteristics, applications, and evaluation measures of chatbots in the field of health [56,92]. In reflection of the findings of these studies, where applicable for the purposes of our study, we chose items from validated tools or items used to evaluate other chatbots (eg, CORE-10, ETQ, AES, and classifier performance measures such as accuracy and precision) to permit cross-comparability where possible between this and existing research and to support efforts to align evaluative measures within the field [56,89,92-94].

### Data Analysis

The effectiveness of the technology will be assessed primarily through data collected via voluntary follow-up surveys and client interactions and engagement with the chatbot. As the use of the chatbot is anonymous, it is not possible to conduct direct follow-up via electronic medical records to confirm the use of any particular service. As such, the research team will primarily rely on information volunteered by clients via the follow-up surveys and chatbot analytics to assess whether clients are successfully connected with recommended resources (by asking them directly).

### Outcome Evaluation

The primary outcome measure will be an analysis of participant responses to follow-up survey questions on a successful connection with resources recommended by the chatbot and the perceived appropriateness of the resources recommended. More specifically, we ask the participants the following questions: (1) “Was/were the resource(s) the MIRA chatbot recommended to you during your conversation appropriate?” (response options: “yes”; “no”; and “other, please specify”) and (2) “After your conversation with the MIRA chatbot, did you follow-up or connect with the resource(s) that MIRA recommended?” (response options: “yes”; “no”; “other, please specify”; and “I prefer not to answer”; if “no,” then the respondent is asked the subsequent question “why not?”).

Assessment of whether the chatbot successfully connected the respondent with appropriate resources will be the number of respondents who answer “yes” to both of these questions. Further consideration will be given to “no” or “other, please specify” responses, where additional detail is shared by the client, to assess rationale for a failed connection (eg, personal choice not to connect or could not successfully reach the resource after attempting to do so). In reflection of previous reports that approximately 24% to 44% of Canadians do not feel that their mental health needs are being adequately met [3,31], we have set a minimum threshold for successful connection with appropriate resources of >50%.

Secondary outcome measures will be an analysis of mental and physical well-being (CORE-10) at the time of use and 24 hours following use, client satisfaction and acceptability (including perceived usability, benevolence, credibility, and trustworthiness), intergroup variation, drop-off and engagement rates, general chatbot use patterns, and exploration of why this intervention may or may not have been supportive or helpful for particular groups.

Chatbot performance will also be evaluated based on additional technical measures identified in reflection of other evaluative works within the field of chatbots in health to allow for the cross-comparability of the findings [56,92,95-97], including an analysis of intent classification accuracy scores, entity recognition accuracy scores, client URL engagement, chatbot rending and response speed, conversational completes, task completion rates measured via binary responses to questions such as “is this what you were looking for?” and “is there anything else I can help you with?,” star ratings by clients at the end of a conversation, client objections, and prompt interruptions.

### Ethics Approval

Ethics approval was granted on August 12, 2021, by the University of Alberta Health Research Board (case Pro00109148) and on April 21, 2022, by the Nova Scotia Health Authority Research Ethics Board (case 1027474). All data and computer code will be password-protected and stored on a secure server at the University of Alberta in Canada.

## Results

On April 1, 2021, this project was initiated by partners MDSC, the University of Alberta, Dalhousie University, the University of Saskatchewan, the International Indigenous Health Research and Training Centre, the Asia-Pacific Economic Cooperation Digital Hub for Mental Health, AI4Society, and the Alberta Machine Intelligence Institute. A Mathematics of Information Technology and Complex Systems Accelerate grant to support student involvement in this project was successfully awarded on August 11, 2021, with a secondary award granted on March 23, 2022, to support research activities for this project until spring 2024. For this study, ethics approval was sought and granted by the University of Alberta Health Research Board and the Nova Scotia Health Authority Research Ethics Board on August 12, 2021, and April 21, 2022, respectively. On May 2, 2022, data collection began and is anticipated to continue until May 2, 2023. Preliminary results will be published in spring or summer 2022, with a more comprehensive evaluation using a larger data set to be completed by spring 2023.

## Discussion

### Overview

The world is undergoing a period of significant growth in technological innovation. Starting with the internet, technological networks and systems have emerged as so complex and disruptive that they have transformed not only our governing and economic structures but also our perception of self, community, and day-to-day life. With 8 million global deaths attributed annually to mental illness [12], there is urgency to identify effective and timely service options that reduce and eliminate barriers, including through health system navigation, as well as to investigate innovations where technology may present constructive, novel solutions.

In this paper, we describe our experience to date with the development of MIRA, a chatbot designed to guide clients who experience mental health challenges to appropriate information and services available to them. Our development process includes a broad team of stakeholders and experts (in mental health and computing science) and addresses a number of challenges that one should consider to develop a realistic and practical solution.

This paper also describes our anticipated methodology to evaluate MIRA, including its ability to connect health care workers and their families with relevant, high-quality mental health services and information. As noted, we hypothesize that the chatbot will effectively connect clients with appropriate information on mental health issues, services, and programs based on personal needs. If proven effective, in the spirit of the Canadian universal health care system, the chatbot will be offered free of charge to the Canadian public. To our knowledge, there have been no similar studies in this field. If successful, this innovation has the potential to offer significant benefits to the Canadian public and demonstrate a solution that can be adopted by other international health care systems.

### Future Directions

There are several considerations that could be made for future research, some of which our team seeks to touch upon in future work related to this study, outlined in this section.

Research evaluating health chatbots is commonly criticized for being inconsistent in terms of outcome measures, which hinders opportunities for cross-comparability with other evaluations. As such, through the careful review of previous studies and the publication of our protocol, we hope to help support a movement toward consistency by using evaluative measures consistent with those reported in systematic and scoping reviews of chatbots in mental health where possible and appropriate [56,92,95-97]. Further consideration should be given by other researchers to the development of a standardized approach to evaluate chatbots within the field of health.

Discussions on the development and implementation of ethical AI and prioritizing health equity throughout the life cycle of an AI system are of critical importance. AI has been criticized for being “no more than human behaviour reflected back to us” [98]. Inherent to this argument is the ability of AI to “reflect the biases present in our collective conscience” [70]. The discourse on guidelines to rectify and prioritize health equity in the development life cycle of an AI system [99], as well as around ethical AI applications generally, is becoming more prolific. This project uses a multidisciplinary core team and advisory committee that includes members of the Indigenous community and other communities of color, individuals with lived experience, and other experts. This cocreation of a mental health chatbot (including efforts to action the First Nations Principles of Ownership, Control, Access, and Possession) with the support of an advisory group to assist usability testing and the development of a controlled training ground truth data set is novel and presents an interesting and rich opportunity to conduct an analysis and exploration of mental health equity in the digital space through a lens of different existing and potential end users. Researchers should be encouraged to continue to explore these topics further in the context of applied AI design and implementation to support health equity and racial justice.

This study will also explore other areas of interest, such as the analysis of health information–seeking behavior (HISB). HISB is a coping strategy individuals use involving the gathering of information about a health topic in response to a recent diagnosis or for other health-related reasons, such as general health promotion. The personal and contextual considerations of HISB have not been adequately explored. More specifically, further analysis is needed of the cultural, contextual, and demographic influences that may play a role in HISB [97,100]. As the perceived level of quality of information accessed can influence individuals’ intention to seek further information, additional considerations must be made for quality review and assurance of any resources recommended by the chatbot. As such, resources that the chatbot recommends must be vetted by experts, including health professionals and individuals with lived experience [97,101,102]. Our team has sought to address this through the development and use of the MIRA Resource Portal and vetting, supported by an Expert Advisory Committee that includes a diverse set of voices. Thus, further analysis of the topic of HISB is possible as a result of this work.

Although deep learning models currently have the ability to conduct language processing tasks such as tagging, text classification, machine translation, and question answering, existing state-of-the-art models are criticized for lacking *explainability*—more specifically, being able to describe how the algorithm came to a particular result or action, which is considered a key pillar in discourse around ethical AI development [103-105]. This and future studies must seek to improve the methods of explainable NLP.

Another direction for future consideration is the incorporation of emotional intelligence into dialogue generation to better imitate human conversational patterns and appropriately respond to emotional input. Existing neural dialogue systems such as sequence-to-sequence have been criticized for being limited in response length or for producing generic or noncommittal versus empathetic or emotionally intelligent outputs [106,107]. Future studies should explore the integration of empathetic response generation that appropriately categorizes a client’s current emotional state based on their input utterance, considers a desired target emotion to guide clients toward, and subsequently generates an emotionally intelligent response back to clients incorporating these considerations [106,107]. Multilabel emotion mining may be considered to support this categorization [108]. Our team will seek to improve the emotional intelligence of future iterations of MIRA following this pilot through the further enhancement of *eEliza*.

### Limitations

There are several anticipated limitations of note that we consider unavoidable. First, digital interventions are not accessible to all Canadians, and there are barriers to their use, including technical issues with connectivity; lack of access to electrical or technological infrastructure because of cost, service provision, and natural disasters; and distrust of technology regarding the use of data or protection of anonymity [11].

Second, consistent adaptation and refinement are inherent in innovations using AI as the technology is programmed to remember interactions with clients and will evolve or learn. In addition, there will be a number of technological bugs or errors in the programming code for the chatbot that will become apparent as it is being piloted. As such, it is anticipated that the technological device itself will require ongoing adaptation with implementation. Any changes observed or made by the study team will be carefully documented and made available upon request.

There is potential for selection bias as participant recruitment includes the use of snowball sampling or chain-referral sampling using the research team’s network or referrals through affiliated organizations (listed with the authors of this paper) to help encourage participant recruitment. In addition, individuals who are more familiar or comfortable with technology may be more likely to participate in this study. To reduce this form of bias, the study team plans to use multiple methods of participant recruitment, including printing hard copies of the study poster for use on bulletin boards in staff rooms in hospitals as well as asking hospital operations staff, with approval from the respective health authorities, to share information about the study with their staff widely.

There is a risk of response bias. As such, our team will seek to oversample in each province; ensure that the anonymity of the survey is clear to the users by outlining anonymity in the welcome message of the chatbot as well as in the consent documentation; primarily use validated tools or items extracted from validated tools to assess baseline mental and physical health as well as user satisfaction and acceptability, including perceived usability, benevolence, credibility, and trustworthiness (ie, the CORE-10, ETQ, and AES); and ensure that the Likert-scale questions include a neutral response option. Data will then be weighted where possible according to the Statistics Canada Census Profile data.

## Appendix

Multimedia Appendix 1 A multidisciplinary approach to mental health chatbot development.

Multimedia Appendix 2 Chatbot storyboard—screenshots of the chatbot interface with details on chatbot functions and features.

Multimedia Appendix 3 Chatbot storyboard—screenshots of the chatbot interface with details on chatbot functions and features.

Multimedia Appendix 4 Chatbot storyboard—screenshots of the chatbot interface with details on chatbot functions and features.

Multimedia Appendix 5 Chatbot storyboard—screenshots of the chatbot interface in eEliza.

## Abbreviations

AES: Acceptability E-scale
AI: artificial intelligence
CORE-10: Clinical Outcomes in Routine Evaluation System
ETQ: Embodied Conversational Agent Trust Questionnaire
HISB: Health information–seeking behavior
MDSC: Mood Disorders Society of Canada
MIRA: Mental Health Intelligent Information Resource Assistant
NLP: natural language processing

## Data Availability

The data sets generated and analyzed during this study are available from the corresponding author upon reasonable request.
