# Supplementary figures and images for: Developing, Implementing, and Evaluating an Artificial Intelligence–Guided Mental Health Resource Navigation Chatbot for Health Care Workers and Their Families During and Following the COVID-19 Pandemic: Protocol for a Cross-sectional Study
Source: JMIR Res Protoc. 2022 Jul 25;11(7):e33717. doi: 10.2196/33717 (PMC9361145; doi:10.2196/33717)

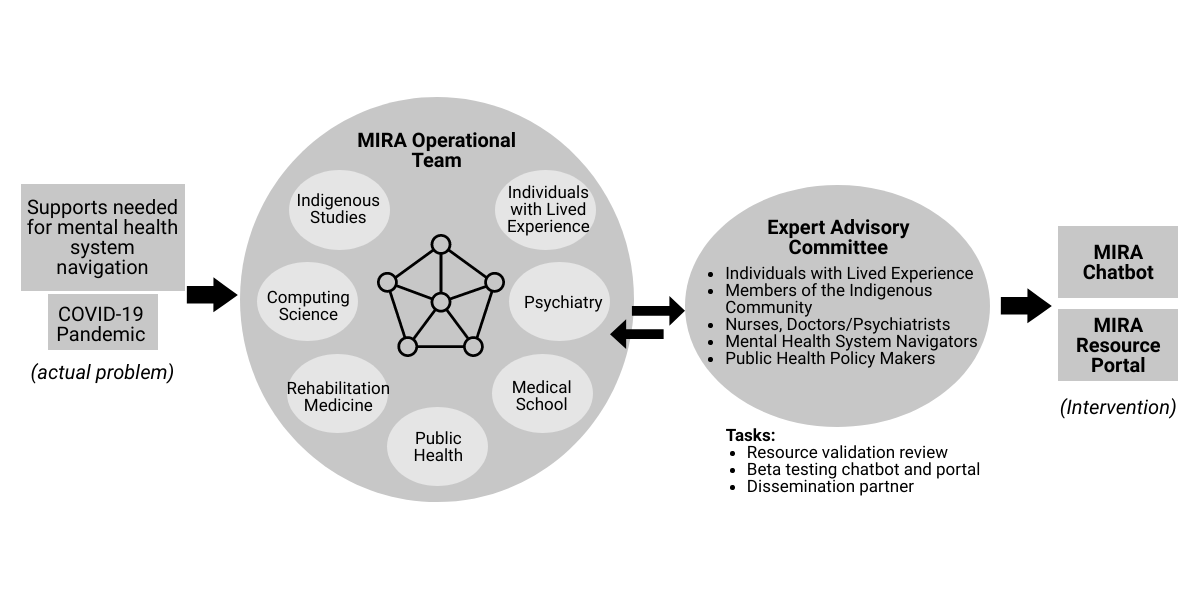

Supplement: Multimedia Appendix 1 [file resprot_v11i7e33717_app1.png]

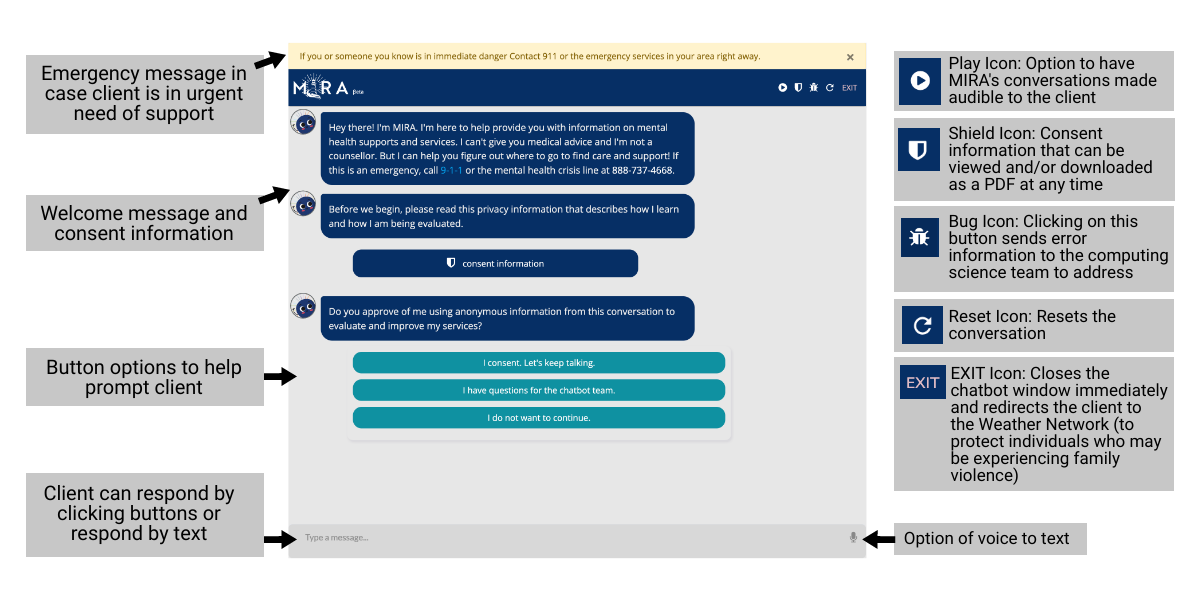

Supplement: Multimedia Appendix 2 [file resprot_v11i7e33717_app2.png]

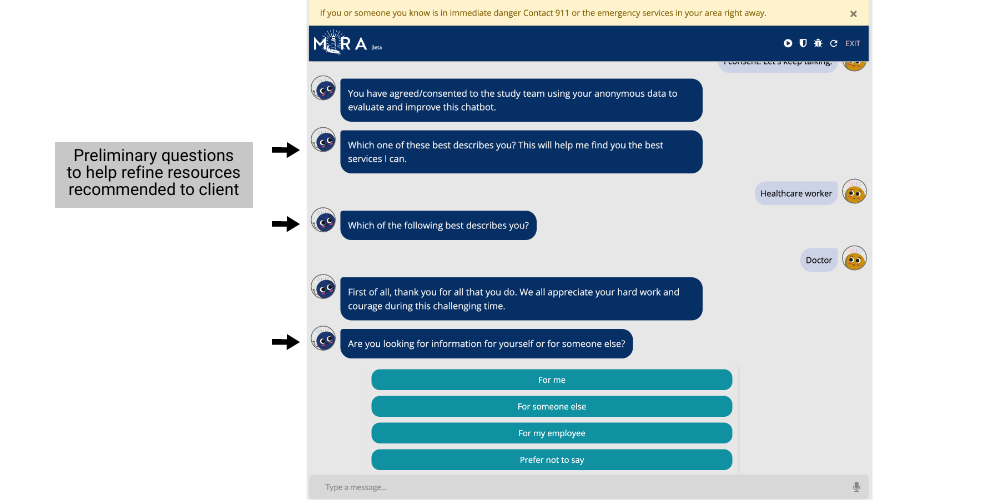

Supplement: Multimedia Appendix 3 [file resprot_v11i7e33717_app3.png]

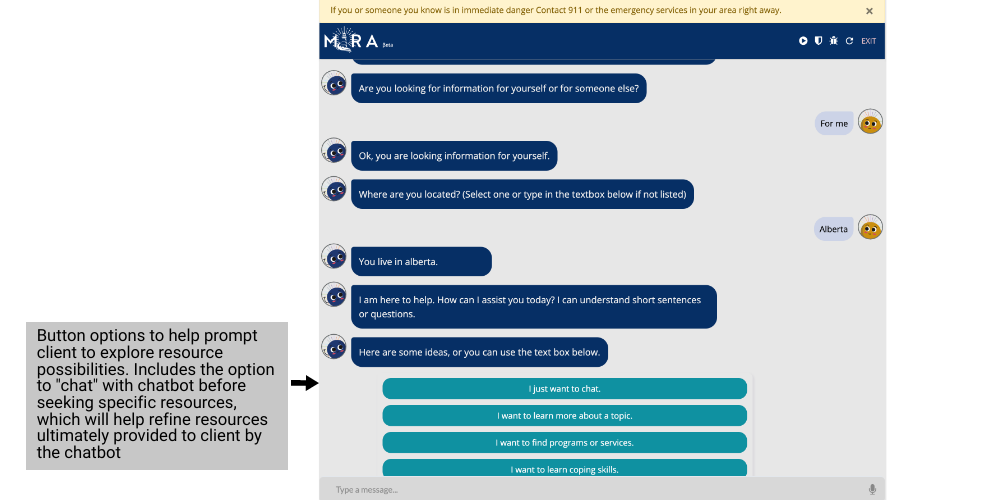

Supplement: Multimedia Appendix 4 [file resprot_v11i7e33717_app4.png]

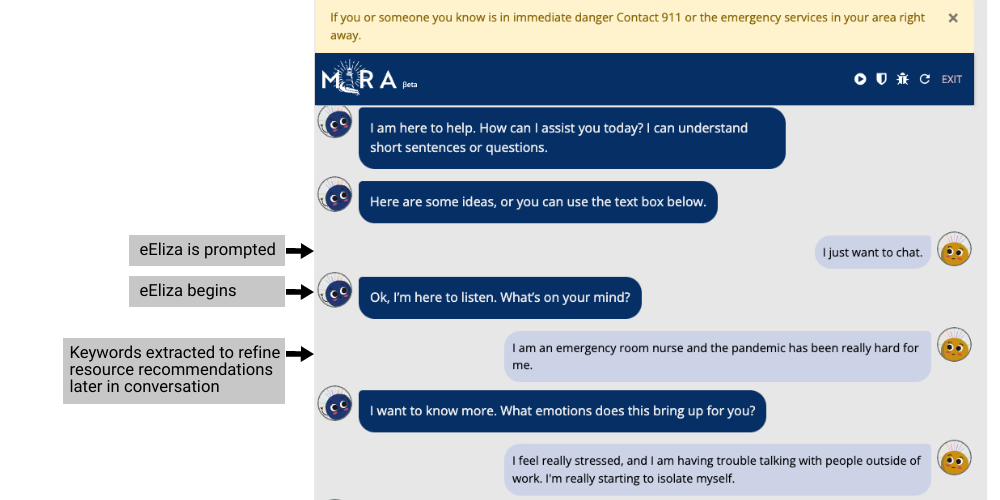

Supplement: Multimedia Appendix 5 [file resprot_v11i7e33717_app5.png]
